# Development of a multiplex detection system for toxic *Amanita* species based on isothermal recombinase polymerase amplification and CRISPR/Cas12b technology

**DOI:** 10.3389/fmolb.2026.1832406

**Published:** 2026-10-12

**Authors:** Hao Li, Xinyu Ying, Zhidan Luo, Chen Lu, Ping Zhang

**Affiliations:** 1 College of Life Sciences, Hunan Normal University, Changsha, China; 2 Jiangsu Key Laboratory of Marine Biological Resources and Environment, Jiangsu Key Laboratory of Marine Pharmaceutical Compound Screening, Jiangsu Ocean University, Lianyungang, China; 3 Co-Innovation Center of Jiangsu Marine Bio-industry Technology, Jiangsu Ocean University, Lianyungang, China

**Keywords:** *Amanita* species, CRISPR/Cas12b, multiplex detection, point-of-need testing, recombinase polymerase amplification

## Abstract

**Background:**

Toxic *Amanita* species are a major cause of fatal mushroom poisoning worldwide. Their morphological similarities to the edible species of mushrooms increase the risk of accidental ingestion, highlighting the need for rapid and accurate identification.

**Objective:**

We aimed to develop a multiplex detection system using isothermal recombinase polymerase amplification (RPA) and CRISPR/Cas12b technology for rapid and specific detection of multiple toxic *Amanita* species.

**Methods:**

Species-specific RPA primers and single guide RNAs (sgRNAs) were designed and optimized for six toxic *Amanita* species (*A. exitialis*, *A. pallidorosea*, *A. subjunquillea*, *A. rimosa*, *A. molliuscula*, and *A. fuligineoides*). The detection system combined RPA with Cas12b-mediated fluorescence readout to evaluate the specificity and sensitivity, and the results were visualized using lateral flow strips and a microfluidic disc chip for parallel detection.

**Results:**

The selected primer–sgRNA combinations showed high specificity values without cross-reactivity and a low detection limit of 10 pg of genomic DNA per reaction. The lateral flow strips enabled intuitive and reliable detection. Furthermore, the microfluidic disc chip enabled multiplex detection for both single-species and four-species mixed samples.

**Conclusion:**

We successfully established a rapid, sensitive, and specific multiplex detection system for toxic *Amanita* species using purified genomic DNA from authenticated specimens, providing a proof-of-concept analytical strategy for informing future development of instrument–light species identification tools in mushroom poisoning cases. We also summarize the evaluations required for further optimization of the current design prior to practical application.

## 1 Introduction

Toxic *Amanita* refers to mushroom species within the genus *Amanita* that contain lethal cyclic peptide toxins. These fungi contribute to more than 90% of fatal cases among individuals poisoned when foraging for wild mushrooms (He et al., 2019). Therefore, toxic *Amanita* is regarded as one of the major public health concerns worldwide. Among the ∼50 highly toxic species of *Amanita* reported globally, 12 types have been identified in China, where they are particularly widely distributed and commonly found in the southern provinces within tropical or subtropical zones (Cui et al., 2023; Huang et al., 2023; Su et al., 2025); these comprise *A. fuliginea* and related *Amanita* species with some of the highest toxicity profiles, including *A. exitialis* (Chen et al., 2025), *A. pallidorosea* (Zhou et al., 2021), and *A. subjunquillea* (Ma et al., 2025). Notoriously, there are remarkable similarities between the lethal and edible species in section *Phalloideae*, such as *A. fuliginea* and *A. caojizong* (Zhang et al., 2015; Cui et al., 2018). Consequently, the risk of inadvertent ingestion and subsequent mortality is higher. Several toxins have been identified in toxic *Amanita* species (Barbosa et al., 2023; Tuğcan and Akpınar, 2025), among which α-amanitin has been highlighted as the culprit in fatal cases (Xue et al., 2023; Wang et al., 2025). The typical symptoms of poisoning develop in four successive stages: incubation, acute gastroenteritis, pseudorecovery, and hepatic/renal failure (Enjalbert et al., 1993; 2002). Thereafter, death may follow opportunistically within several days. Fortunately, the effectiveness of immediate targeted treatment has been proven for rescuing organ damage and preventing death subsequent to ingesting toxic *Amanita* (Li Y. et al., 2018). Rapid identification of toxic *Amanita* species and their associated toxins is therefore a pivotal requirement for responding within the available treatment window. Immunoassays have advanced amanitin detection by enabling sensitive measurement of β-amanitin in human serum and urine (Abuknesha and Maragkou, 2004). However, antibody cross-reactivity can complicate discrimination among related toxins, and toxin detection alone does not establish the identity of the causative mushroom species.

Identification methods based on isothermal amplification perfectly match this demand compared to other methodologies of detecting toxic *Amanita*. Recombinase polymerase amplification (RPA) is an isothermal nucleic acid amplification technique performed in the temperature range of 37 °C–42 °C, where the reaction energy is supplied by the adenosine triphosphate and creatine phosphate regeneration system (Piepenburg et al., 2006). Compared to DNA barcode technology, isothermal amplification eliminates the requirement for sequencing and subsequent complex bioinformatics analysis. Furthermore, unlike traditional polymerase chain reaction (PCR) coupled with gel electrophoresis or real-time quantitative PCR (qPCR), a thermal cycler or postamplification electrophoresis is not required for RPA (Gao et al., 2022; He et al., 2026). Consequently, the equipment cost and operational complexity are reduced significantly.

RPA and loop-mediated isothermal amplification (LAMP) are two popular isothermal amplification technologies available owing to their distinct primer design strategies and enzymatic reaction principles. The cost, speed, sensitivity, and specificity are very similar between RPA and LAMP. However, the reaction temperature for RPA is in the range of 37 °C–42 °C, which is much lower than that of LAMP (Lobato and O’Sullivan, 2018). Moreover, RPA was proven to have higher tolerance to potential inhibitors in complex samples, allowing minimization of the sample purification requirements (Kersting et al., 2014). These properties make it easier for RPA to provide rapid on-site testing with complex samples, such as vomit or partially digested food from poisoned patients. Despite these advantages, RPA performance can be affected by reaction conditions and mixing, underscoring the need for careful assay optimization (Lillis et al., 2016). Additionally, CRISPR/Cas technology has been combined with RPA, LAMP, and other isothermal amplification technologies and shown to offer remarkable specificity enhancement (Gootenberg et al., 2017; Li S. Y. et al., 2018; Li et al., 2019) in the identification of various pathogenic microorganisms (Sam et al., 2021; Aman et al., 2022; Wu et al., 2023). In an earlier study by our team, *A. fuliginea* was effectively distinguished from a panel of toxic *Amanita* using an assay combining RPA with CRISPR/Cas12b. For simple and quick use of this assay, colloidal gold antibody lateral flow strips (LFSs) were fabricated and showed qualified performance.

Numerous species of toxic *Amanita* have been found globally and are widely spread throughout China. Moreover, overlapping spatiotemporal distributions have been observed frequently among toxic mushrooms, particularly in the boom season. This could increase the risk of ingesting multiple suspected species in a single incident and the uncertainty in identifying the exact species from a panel of candidates. If the detection is limited to a single species, then the diagnostic efficacy is restrained and could lead to a lapse in the treatment time window. Given this dilemma, a high-throughput detection strategy is required and could be met with the chip-based approach. Using pre-deposited assays aimed at identifying multiple toxic mushrooms, we can distinguish a panel of suspected targets parallelly through a single round of reaction. Moreover, other advantages are available with the chip-based strategy, including high sensitivity, miniaturization, and automation. This chip-based approach can enable rapid and accurate identification of the species involved in accidental ingestion of toxic mushrooms, which may in turn inform subsequent treatments targeting the distinct toxin poisoning mechanisms underlying divergent toxic *Amanita*. In the present study, we designed an RPA-CRISPR/Cas12b system and validated it for six kinds of toxic *Amanita* in addition to *A. fuliginea*, namely, *A. exitialis*, *A. pallidorosea*, *A. subjunquillea*, *A. rimosa*, *A. molliuscula*, and *A. fuligineoides*. Subsequently, a microfluidic disc chip trial (Li et al., 2026; Long et al., 2026) was conducted to integrate the assays for four targets, including the one developed in our earlier study and three others generated in the current work, and a multiplex detection scheme was achieved effectively for the selected species. In the present study, the assay was extended from a single species in a multiplex manner and integrated into a ready-to-use microfluidic disc chip for parallel detection from a single input sample, surpassing our earlier work.

## 2 Materials and methods

### 2.1 Preparation of genomic DNA from toxic *Amanita* samples

Toxic *Amanita* samples were collected from multiple sites in China; they were subsequently identified and preserved by the Fungal Research Laboratory, College of Life Sciences, Hunan Normal University. In this work, seven species were included and are listed in Table 1, along with their accession numbers for the genomic DNA sequences and information about sample collection, including time, location, and preservation ID.

**TABLE 1:** Species of mushrooms used in this study.

Species (Latin name)	GenBank accession number	Sampling time	Sampling location	Preservation ID
*Amanita fuliginea*	KU601412	6 June 2017	Dazhongqiao, Qiyang County, Yongzhou City, Hunan province	MHHNU9047
*Amanita exitialis*	AY855212	21 March 2009	Baiyunshan, Guangzhou City, Guangdong province	MHHNU30297
*Amanita pallidorosea*	FJ176736	26 June 2020	Siqu, Yanhe County, Tongren City, Guizhou province	MHHNU10353
*Amanita subjunquillea*	MH508624	28 August 2020	Baijifeng, Tonghua County, Tonghua City, Jilin province	MHHNU10524
*Amanita rimosa*	NR_119714	9 June 2017	Shangyunqiao, You County, Zhuzhou City, Hunan province	MHHNU9050
*Amanita molliuscula*	NR_147633	6 August 2017	Changbaishan, Fusong City, Jilin province	MHHNU9142
*Amanita fuligineoides*	KC755030	6 September 2007	Mangshan, Yizhang County, Chenzhou City, Hunan province	MHHNU7164

The genomic DNA samples were then extracted using E.Z.N.A.® Plant DNA DS Kit (catalog no. D2411-01, Omega Bio-tek). To minimize the risk of cross-contamination, the DNA extraction and preamplification processes were performed in a dedicated preamplification room that was physically separated from the postamplification detection area. For sample preparation, 20 mg of dried tissue from each species was ground using a disposable Kontes pestle to achieve a fine powder. The powdered samples were then processed as per the manufacturer protocol supplied with the kit. To remove RNA contamination, on-column digestion was carried out with RNase A. The quality and yield of the DNA product were then determined using a NanoDrop spectrophotometer to assess concentration, A_260_/A_280_, and A_260_/A_230_ values. Lastly, samples with both A_260_/A_280_ > 1.8 and A_260_/A_230_ > 2 were selected for the following assays.

### 2.2 Design of primers and single-guide RNAs (sgRNAs)

The primer pairs used in the species-specific RPA reactions were designed online using the NCBI Primer-BLAST tool based on the 5.8S ribosomal RNA gene and internal transcribed spacer (ITS) 1/2 of the corresponding species. The secondary structures of the primers were analyzed online using Millipore OligoEvaluator™ (https://www.oligoevaluator.com/LoginServlet). If strong secondary structures were predicted, point mutations were introduced on the 5′ side of the stem region (Li et al., 2025). Oligonucleotide synthesis was conducted commercially (General Biol (Anhui) Corp., Ltd.).

The sgRNA sequences were designed within the amplified regions of the RPA reactions. In each region, the Cas12b protospacer adjacent motif (PAM) sequence (TTN) was identified first, and the adjacent 20 bp sequence downstream of the selected PAM was obtained as the target sequence of the candidate sgRNA. The sgRNAs can be acquired from either strand of the DNA.

All designed candidate primer pairs are listed in Table 2, and the candidate sgRNAs are given in Table 3.

**TABLE 2:** Sequences of the primer pairs designed for this study.

Species	No.	Sequence (5’-3’)	GC%	Length	Amplicon size (bp)
*Amanita fuliginea*	5F	ACTTTCAACAACGGATCTCTTGGCTCTCGC	50.00%	30	361
	5R	TTGGTCAAATGAGGTTCAAACTGGACATGG	43.33%	30	
*Amanita exitialis*	1F	TGGCATTTTATTAGACACAAGGTCCGAGAT	40.00%	30	409
	1R	CCATTTTTACAGACAGTTAGACAGCAGAGA	40.00%	30	
	2F	TGGCATTTTATTAGACACAAGGTCCGAGAT	40.00%	30	411
	2R	GTCCATTTTTACAGACAGTTAGACAGCAGA	40.00%	30	
	3F	GTCCATTTTTACAGACAGTTAGACAGCAGA	40.00%	30	496
	3R	CCTGTGCACACTTGTAGACACTTGGGAATG	50.00%	30	
	4F	ATACATAATACAAGTTTCAACAACGCATCA	30.00%	30	357
	4R	GTCCATTTTTACAGACAGTTAGACAGCAGA	40.00%	30	
*Amanita pallidorosea*	1F	TGTAGACACTTGGGACTGAGAGACTTTGAC	46.67%	30	441
	1R	ACTTGATGTTGCTCCTGGCATTGATAGATT	40.00%	30	
	2F	AACTTTCAACAACGGGTCTCTTCGCTCTCG	50.00%	30	211
	2R	GCCTGTAACTCCCAAAATCCAAVACCTATC	43.33%	30	
	3F	AACTTTCAACAACGCATCTCTCAGCACTCG	46.67%	30	213
	3R	CAGCCTACAACTCCCAAAATCCAATACCTA	43.33%	30	
	4F	AACTTTCAACAACGCATCTCTCGGCTCTCG	50.00%	30	215
	4R	AACATCCTACAACTCCCAAAATCCAATACC	40.00%	30	
*Amanita subjunquillea*	1F	TAAAATCTACCTTACATCTTTCAACAACAG	30.00%	30	383
	1R	CCATCCATACTACTTACTACAGACCGACAC	46.67%	30	
	2F	AAACATAGAGAACGATTTTCAACAACGGAT	33.33%	30	365
	2R	ATACAGACAGTCACAGTTAGACAGCAGAGA	43.33%	30	
	3F	AAAAATACCCTACAAGTTACAACAACGGAT	33.33%	30	363
	3R	AGACAGTCACAGTTAGACAGCAGAGAGAAG	46.67%	30	
	4F	TACAAGTTTCAACAACGGTTCTCTTCAGTC	40.00%	30	289
	4R	ATCACACCAATGGAGTTCAATGGCTTTTCT	40.00%	30	
*Amanita rimosa*	1F	GAGACTTTAACCAGCCCCTTGAGAAATTGAA	41.94%	31	316
	1R	CCCCATAAATCCGAAACCTATCAAAAGCAGA	41.94%	31	
	2F	GACTTTAACCAGCCCCTTGAGAAATTGAACT	41.94%	31	429
	2R	TTCATTGCTCCTGGCATTGAGACCATTTTAT	38.71%	31	
	3F	TAACCAGCCCCTTGAGACATTGAACTTGCCA	48.39%	31	431
	3R	TGGTATCAATGCCAGGAGCAATGAAGAACAG	45.16%	31	
*Amanita molliuscula*	1F	CTCTTGAGGAGTTGAAATCTGGGTGTCTATG	45.16%	31	375
	1R	GAGTTTCAAATGGCTTTCATCTCCACTAATA	35.48%	31	
	2F	CTTGAGGAGTTGAAATCTGGGTGTCTATGCC	48.39%	31	375
	2R	TGGCGGTTCAAATGGCTTTCATCTCCACTAA	45.16%	31	
*Amanita fuligineoides*	1F	TCTGGGACTCTATGCCATTTTATTAAACACT	35.48%	31	279
	1R	CCCCAAAATCCAATACCTATCAAGTTCAGAC	41.94%	31	
	2F	TGTCGCTGTCCCATCTGTGCATGTGTACGTC	54.84%	31	394
	2R	CCAAAATCCAATACCTATCAAATTCAGACAG	35.48%	31	

The primer pair sequences of *Amanita fuliginea* were optimized and adopted from Li et al. (2025).

**TABLE 3:** Sequences of the sgRNAs designed for this study.

Species	No.	Sequence (5’-3’)	Strand orientation
*Amanita fuliginea*	1#	AGUGUCAUUAACAUCUCAAU	(+)
	2#	AUGGGUAUUGGAAAUUGGGG	(+)
	3#	CAGGGCUUGUAUAGCUUUGC	(+)
	4#	GUGGAGAAAAAGAAAGCUCA	(+)
*Amanita exitialis*	1#	AGUGUCAUUAAAGUCUCAAG	(+)
	2#	CAGGCUUUUUCAGACCGCCU	(+)
	3#	GUGGAGAAAAAGCCAUUUGA	(+)
*Amanita pallidorosea*	1#	AAUAUAUAAAUACAACUUUC	(+)
	2#	AGUGUCAUUAACACCUCAAG	(+)
	3#	AUAGGUAUUGGAUUUUGGGA	(+)
*Amanita subjunquillea*	1#	AGUGUCAUUAAAUUCUCAAG	(+)
	2#	CAGGCUGUUUCAAAUAAAUA	(+)
	3#	GUGGAGAAAAGCCAUUGAAC	(+)
*Amanita rimosa*	1#	UGGUCAAAGUCUCUCAUUCC	(–)
	2#	CACCCAGAUUUCAAUUCUCU	(–)
	3#	AACAUGCAACUAGUGUUUGG	(–)
	4#	GUUGUAUUUAUAUAUUCAAU	(–)
	5#	AGGCAUGCUCCUCGGAAUGC	(–)
*Amanita molliuscula*	1#	CAACUCCUCAAGAGACUGGU	(–)
	2#	GACAUGCAACUAGGUUUUGA	(–)
	3#	UUGUAUUUUUACAUAUAAAU	(–)
	4#	CAAAGAUUCGAUGAUUCACU	(–)
	5#	UGAGACUUUUAAUGACACUC	(–)
*Amanita fuligineoides*	1#	UUCUCUCAAGAGACCGGGUU	(–)
	2#	UAUGCAACUAGUGUUUAAUA	(–)
	3#	CAUCAUUCUAUAGACAUAUG	(–)
	4#	GUUGUACUUUAUAUACAAUC	(–)
	5#	UUGAGAUUUCAAUGACACUC	(–)
	6#	UCAGACAGGCCUUGAGAUUU	(–)

sgRNA sequences of *Amanita fuliginea* was optimized and adopted from Li et al. (2025).

### 2.3 *In vitro* preparation of sgRNAs

By including a 20 bp T7 promoter at the 5′ end of a candidate sgRNA sequence, the sgRNA (20 bp) in the middle, and a 91 bp sgRNA stem-loop scaffold at the 3′ terminus, we generated a 131 bp sequence and inserted into the pBAD vector *in silico*. The resulting plasmid was designated as pBAD-T7-sgRNA and synthesized commercially. The fragment containing the sgRNA sequence was then amplified from the pBAD-T7-sgRNA plasmid using the PCR primer pair listed in Table 4.

**TABLE 4:** Primer pair to amplify the sgRNA template.

Primer name	Sequence (5′–3′)
131-dsDNA-F	TAATACGACTCACTATAGGGGTCT
131-dsDNA-R	TCCCAAAATCCAATACCTATGTGCCACTTCTCAGATTTGAGA

Next, a 50 μL PCR step was set up for each candidate sgRNA using the 2× Taq-PLUS PCR Mix (BestEnzymes, China), with an annealing temperature of 58 °C and 15 s of elongation for 35 cycles. The obtained 131 bp fragment was then cleaned with a PCR purification kit (Vazyme International, China) and used as the template to generate the sgRNA. *In vitro* transcription of the sgRNA was performed using T7 RNA polymerase (BestEnzymes, China) in a 20 μL reaction maintained at 37 °C for 16 h. To remove the template DNA, the crude products were treated with DNase I at 37 °C for 15 min, followed by the addition of 3 μL of 0.5 M EDTA and incubation at 75 °C for 10 min. After further purification with the SPARKeasy RNA fast purification kit for micro samples (catalog no. AC1001, Sparkjade Scientific, China), the sgRNA was subjected to agarose gel electrophoresis for quality control. We observed sharp bands located slightly higher than the 100 bp marker band, indicating good quality of the sgRNA. Lastly, qualified sgRNA products free of DNA contamination were obtained and used in the CRISPR assays.

### 2.4 Setup of RPA-CRISPR/Cas12b one-pot assay

For each mushroom species, the primer pairs and candidate sgRNAs were combined one-to-one and tested using RPA-CRISPR/Cas12b one-pot assays. The dosage and concentration of all reactive components are listed in Table 5. For each combination, a 25 μL reaction was set up as shown in Table 5 and subjected to quantitative real-time PCR (Hongshi Medical, China) as a program of 30 s/cycle at 40 °C for 60 cycles. The fluorescence intensity was recorded and used to generate curves. For each assay, three technical replicates were performed per DNA sample, and three independent samples were tested per species. A no-template control (NTC) sample was included in each assay using DNase/RNase-free water of the same volume instead of the DNA sample. For each species, we tested all possible combinations of the designed primer pairs and sgRNAs. Based on the final fluorescence intensities generated across these parallel reactions, the combination yielding the highest value was selected as the optimized assay.

**TABLE 5:** Composition of the RPA-CRISPR/Cas12b one-pot assay.

Component	Final concentration or volume
Lyophilized basic iso-amplification mix (catalog no. EG23103S, BestEnzymes, China)	1 granule
Forward primer	0.4 μM
Reverse primer	0.4 μM
Magnesium acetate	14.5 mM
AapCas12b (BestEnzymes, China)	30 nM
sgRNA	210 nM
Single-stranded DNA probe (5′FAM-TTATT-BHQ1-3′)	400 nM
RNase inhibitor (BestEnzymes, China)	20 U
Genomic DNA	2 μL (commonly 100 ng or as indicated by case)
1× Cas12 buffer	Fill to 25 μL
Total volume	25 μL

Alternatively, to test the products on the CRISPR/Cas12b LFSs with a double antibody sandwich (BestEnzymes, China), the reactions were formulated as in Table 5 using the same component doses as the fluorescence-based assays except for the probe. For LFS detection, the single-stranded DNA reporter probe was synthesized using the same sequence by labeling the 3′ end with a biotin group instead of BHQ1. The RPAs were carried out at 40 °C for 40 min. Once the reaction was complete, the products were diluted with DNase/RNase-free water to obtain 10-fold volume. The strips were then dipped in the diluted RPA products and incubated for 5 min at room temperature to observe the bands. In each strip, one control band was prepared at the handling end and one test band was located at the sampling end. A valid test was defined as one showing a clearly visible control band. The results were then determined visually based on the appearance of the test bands in the valid strips.

### 2.5 Microfluidic chip-based detection

To develop the microfluidic chip-based detection system, the chip was designed first by establishing the sample inlet chamber at the center of the chip. Around this chamber and connected to it, several amplification chambers were arranged radially and independently of each other. Microchannels were fabricated for the connections between the sample inlet and amplification chambers.

In each amplification chamber, a single optimized RPA-CRISPR/Cas12b assay was compressed in a lyophilized form before use. In brief, the reagent mixture for the assay was filled in the amplification chamber, and the entire chip was immersed in liquid nitrogen (−196 °C) to instantly form fine ice crystals and maximally preserve the bioactivity. The frozen chip was then transferred to a freeze-dryer for primary drying under vacuum and subsequently subjected to desorption drying by gradually increasing the temperature to remove residual water. The assays in the chip were thus converted into lyophilized cakes with ready-to-use porous structures.

To initiate detection, the DNA solution (prepared from either single or multiple species) was loaded into the sample inlet chamber and pushed to all amplification chambers via centrifugation no higher than 3,000 rpm for 5 min. In each amplification chamber, approximately 25 μL of the reaction mixture was reconstituted and processed. This program was set to 45 s/cycle at 40 °C for 60 cycles to collect the fluorescence signals. The detection was carried out using the RTisochip™ isothermal amplification analyzer, and the fluorescence intensity threshold was defined using the default instrument settings.

To avoid cross-contamination, all reagents were prepared in a laminar flow hood using disposable plasticware. The closed-tube one-pot design of the RPA-CRISPR/Cas12b assay further reduces carry-over contamination risk by eliminating the need to open reaction tubes between the amplification and detection steps. NTCs were included in every batch of assays to monitor for contamination.

## 3 Results

### 3.1 Optimal primer–sgRNA combination was identified from RPA-CRISPR/Cas12b assay

The overall workflow of this study, from sample collection, DNA extraction, RPA, and CRISPR/Cas12b detection to result interpretation via LFS or microfluidic disc chip, is summarized in Figure 1A.

**FIGURE 1:**
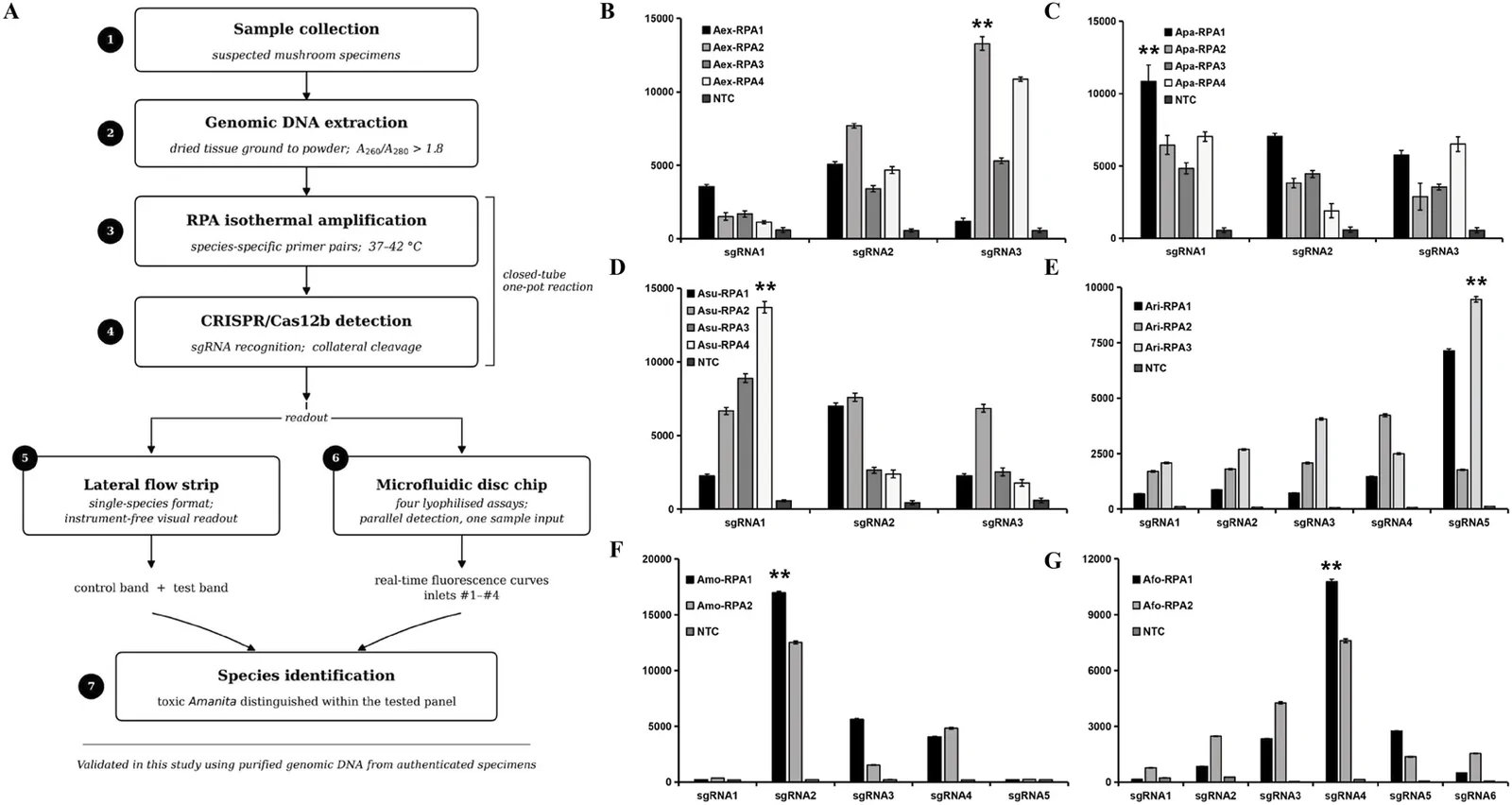
Fluorescence signal intensities of the candidate primer–sgRNA combinations. **(A)** Workflow schematic of the present study. Four pairs of recombinase polymerase amplification (RPA) primers and three single guide RNAs (sgRNAs) were each tested for **(B)** *Amanita exitialis*, **(C)** *Amanita pallidorosea*, and **(D)** *Amanita subjunquillea*. **(E)** Three pairs of RPA primers and five sgRNAs were tested for *Amanita rimosa*. **(F)** Two pairs of RPA primers and five sgRNAs were tested for *Amanita molliuscula*. **(G)** Two pairs of RPA primers and six sgRNAs were tested for *Amanita fuligineoides*. The data are expressed as mean ± standard deviation for n = 3. A combined no-template control (NTC) was included with all experimental groups. The significance was determined by one-way ANOVA. *p < 0.05, **p < 0.01.

To obtain the maximum signal intensity, all candidate primer pairs and sgRNAs were tested in all possible combinations for each species. All combinations were tested in the RPA-CRISPR/Cas12b assay to react with genomic DNA extracted from corresponding mushroom samples. The signal intensity was recorded periodically during the reaction, and bar charts were generated for each species based on the signal captured at the endpoint (Figures 1B–G).

This assessment was carried out for six species, including *A*. *exitialis* (Figure 1B), *A*. *pallidorosea* (Figure 1C), *A*. *subjunquillea* (Figure 1D), *A*. *rimosa* (Figure 1E), *A*. *molliuscula* (Figure 1F), and *A*. *fuligineoides* (Figure 1G). The fluorescence intensities varied considerably among the combinations; however, the optimized combinations (Table 6) reached an intensity level of approximately 10^4^ and were used for all subsequent analyses.

**TABLE 6:** Summary of optimal primer–sgRNA combinations.

Species	Optimized combination	
	Primer pair	sgRNA
*Amanita exitialis*	2#	3#
*Amanita pallidorosea*	1#	1#
*Amanita subjunquillea*	4#	1#
*Amanita rimosa*	3#	5#
*Amanita molliuscula*	1#	2#
*Amanita fuligineoides*	1#	4#

### 3.2 Selected primer–sgRNA combinations showed excellent cross-species specificity

To exclude the potential risk of cross-reactivity between the selected primer–sgRNA combination and non-target DNA samples, we established RPA-CRISPR/Cas12b assay panels. In each panel, one selected primer–sgRNA combination was reacted with genomic DNA derived from both target and non-target species. In the resulting real-time fluorescence signal curves (Figure 2), robustly elevated signals were captured without exception for all assays composed of the primer–sgRNA combinations and target DNA samples. The intensities at the endpoint (60 cycles) were generally in the range of 1 × 10^4^ to 2.5 × 10^4^. In contrast, the curves generated with the primer–sgRNA sets and non-target DNA samples were restricted to the range of 1 × 10^3^ to 2 × 10^3^. The resulting differences between the target and non-target reactions were distinct enough to highlight the correct species of toxic mushroom from similar varieties.

**FIGURE 2:**
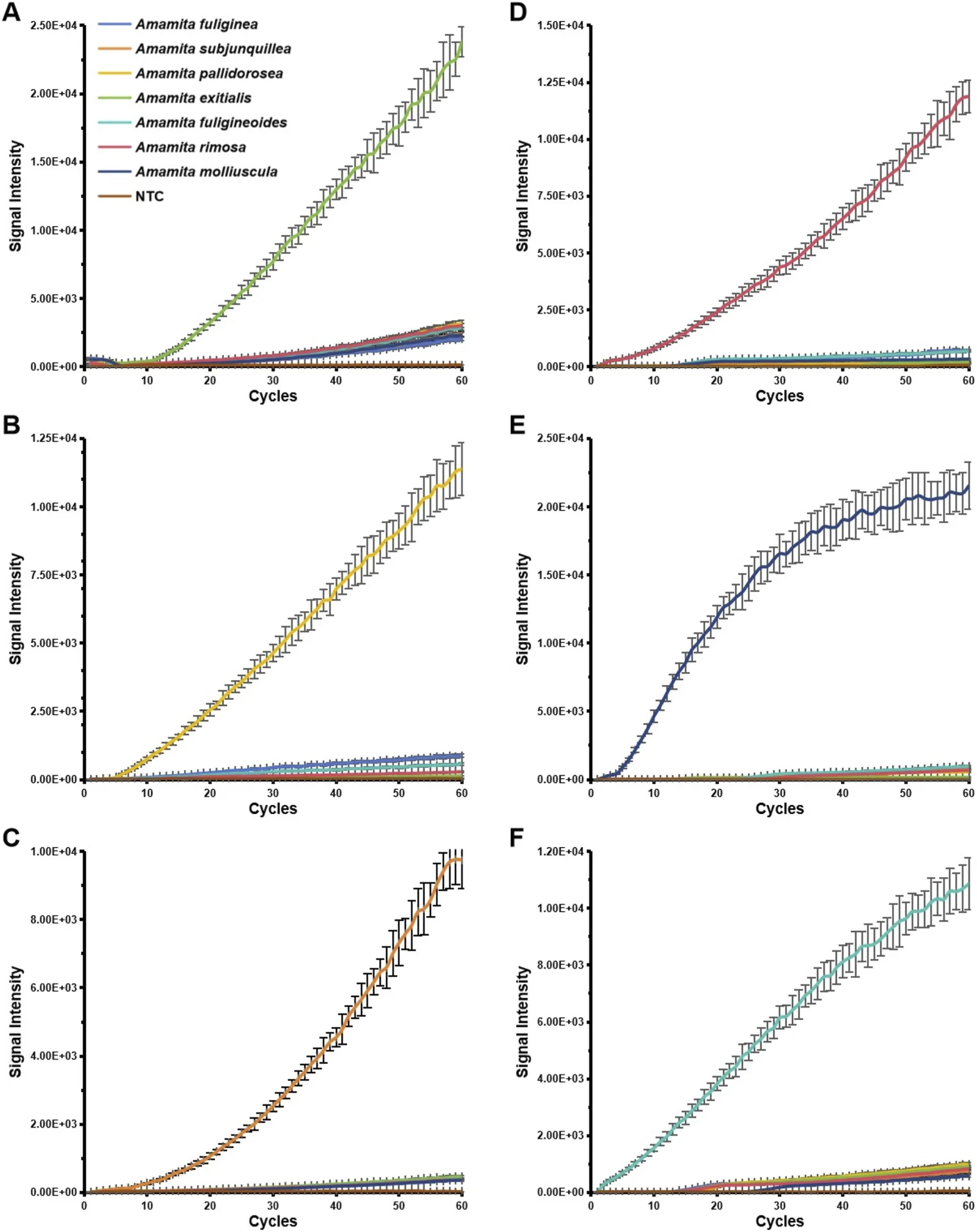
Real-time fluorescence intensity curves to validate specificity. The fluorescence signals were captured from reactions using the optimal primer–sgRNA combinations designed for **(A)** *A. exitialis*, **(B)** *A. pallidorosea*, **(C)** *A. subjunquillea*, **(D)** *A. rimosa*, **(E)** *A. molliuscula*, and **(F)** *A. fuligineoides*. Each combination was reacted with DNA samples extracted from the seven toxic *Amanita* species listed in Table 1.

### 3.3 Selected primer–sgRNA combinations showed remarkable sensitivity in assays

To evaluate the detection capacities of the selected primer–sgRNA combinations for targets of varying abundances, additional RPA-CRISPR/Cas12b assay panels were established. Varying amounts of target genomic DNA, ranging from 10 pg to 100 ng per reaction, were reacted with the corresponding primer–sgRNA combination in each panel. As visualized in the real-time curves (Figure 3), the final fluorescence intensities were positively correlated with the input doses of the target DNA samples. Moreover, even at input DNA doses as low as 10 pg per reaction, the resulting signals and curves were clearly detectable and different from those of the negative control, meaning that the assays can be performed effectively with DNA samples as low as 10 pg/reaction.

**FIGURE 3:**
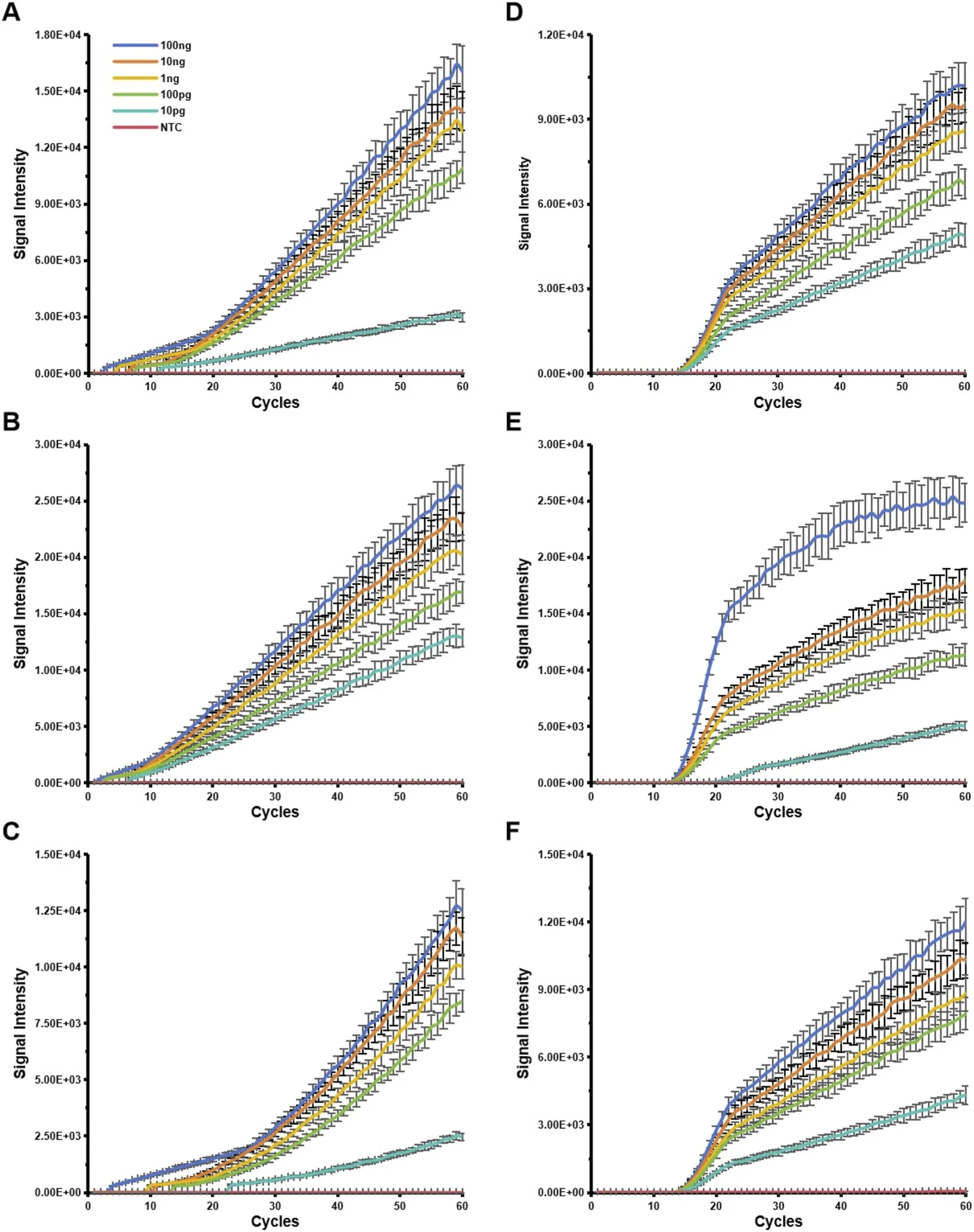
Real-time fluorescence intensity curves to reveal sensitivity. The fluorescence signals were captured from reactions using the optimal primer–sgRNA combinations for **(A)** *A. exitialis*, **(B)** *A. pallidorosea*, **(C)** *A. subjunquillea*, **(D)** *A. rimosa*, **(E)** *A. molliuscula*, and **(F)** *A. fuligineoides* with target DNA sample doses ranging from 10 pg to 100 ng per reaction.

### 3.4 Toxic *Amanita* were distinguished via LFSs following RPA-CRISPR/Cas12b assays

To present the results of the assays in an intuitive manner, LFSs were used for Cas12/13 detection. After the reactions, the LFSs were dipped in 10-fold diluted samples for 5 min. On each strip, one control band and one test band were observed; in each panel, the control line emerged clearly (dashed box), confirming the validity of strip-based detection. The test band (red arrowhead) was only visible when the assay was carried out with the indicated primer–sgRNA combination and its corresponding DNA sample, while no bands were detectable for the non-corresponding DNA samples (Figure 4).

**FIGURE 4:**
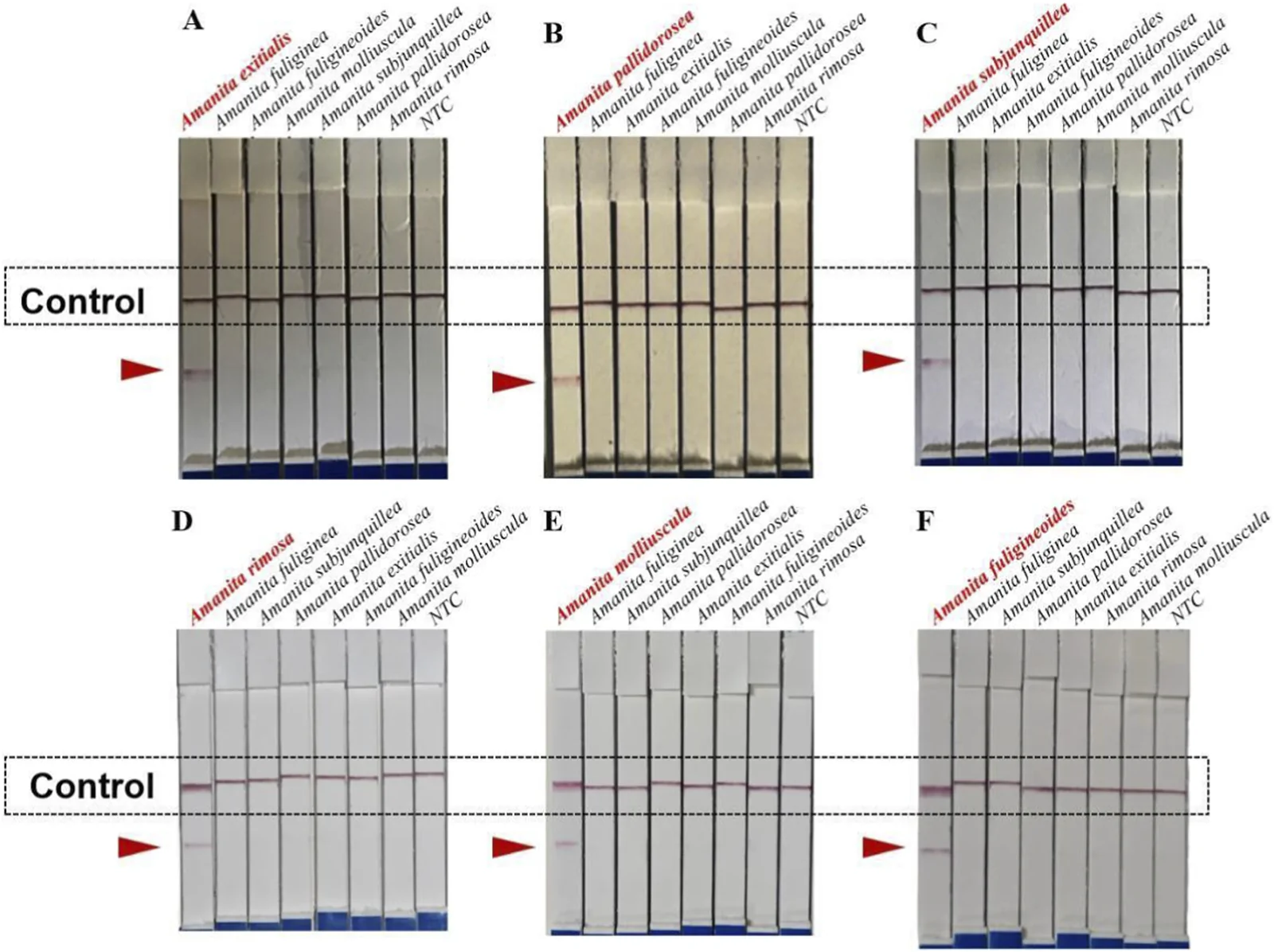
Images of lateral flow strips visualizing the results of the RPA-CRISPR/Cas12b assays. The panel assays using the strips were carried out for **(A)** *A. exitialis*, **(B)** *A. pallidorosea*, **(C)** *A. subjunquillea*, **(D)** *A. rimosa*, **(E)** *A. molliuscula*, and **(F)** *A. fuligineoides* with a set of target DNA samples. The control bands on the strips are shown with the dashed boxes, while the target detection bands are shown with red arrowheads.

### 3.5 RPA-CRISPR/Cas12b assay on chip was established for parallel detection

To identify the exact toxic *Amanita* species from a panel of suspected candidates, preliminary tests were performed using disc chips (Figure 5A) containing specific assay targets; the sample inlets numbered 1–4 were loaded with individual assays targeting *A. fuliginea, A. pallidorosea, A. subjunquillea*, and *A. exitialis*, respectively. Each chip was loaded with a single DNA sample or mixture to all inlets deposited with assays.

**FIGURE 5:**
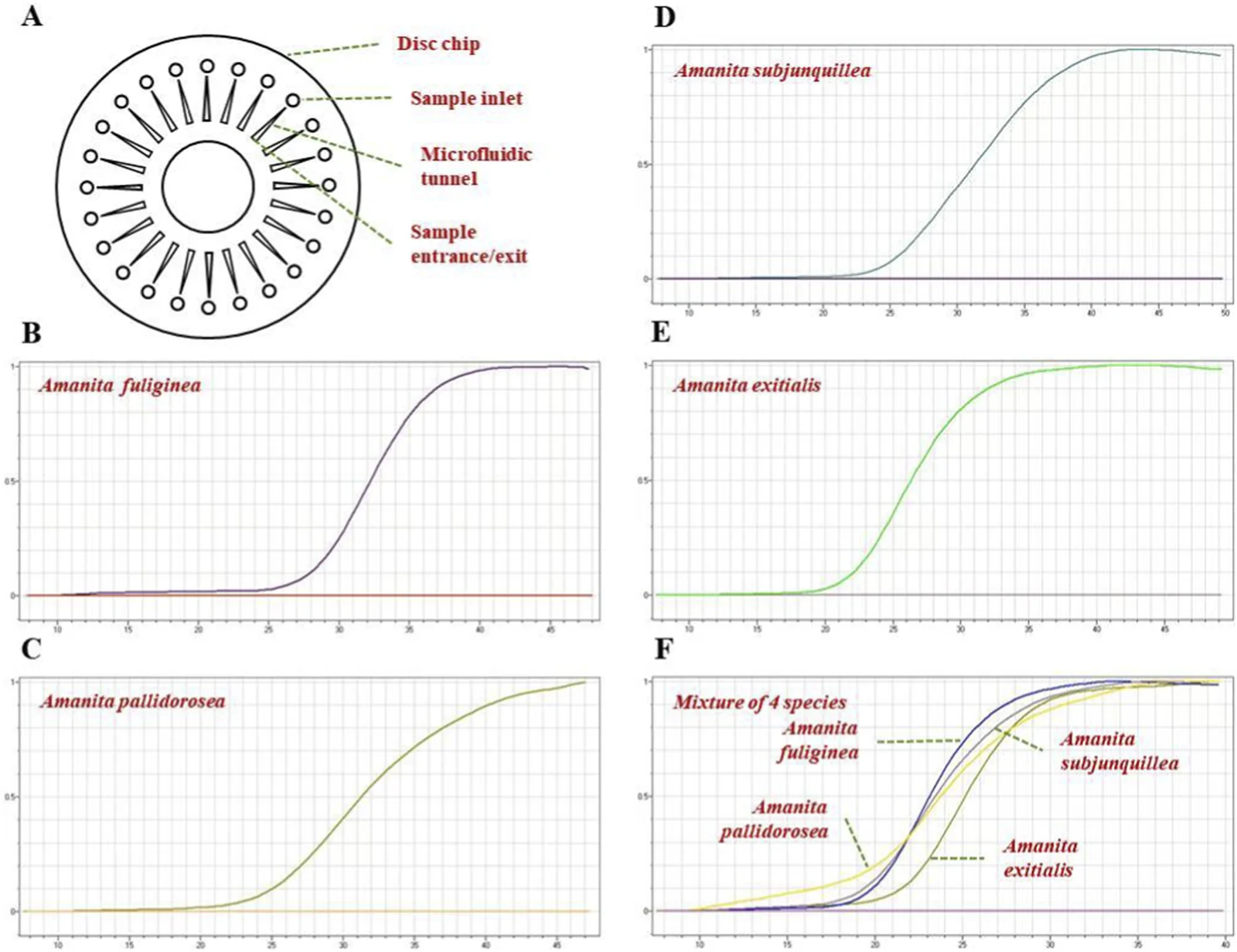
Relative fluorescence intensity curves generated with on-chip reactions. **(A)** Schematic illustration of the microfluidic disc chip manufactured for parallel RPA-CRISPR/Cas12b assays. Curves captured on-chip using DNA samples of **(B)** *A. fuliginea*, **(C)** *A. pallidorosea*, **(D)** *A. subjunquillea*, **(E)** *A. exitialis*, and **(F)** mixture of these four species.

Consistent with the specificity demonstrated in Figures 2, 4, the relative fluorescence intensity curves were recorded only at inlets reacting to their corresponding DNA samples (Figures 5B–F). Lastly, signal curves were generated simultaneously at inlets 1–4 when the mixed DNA sample was loaded.

## 4 Discussion

Owing to the widely observed high mortality rate (Trakulsrichai et al., 2017), quick progression of poisoning (Enjalbert et al., 2002), and shared spatiotemporal cross-species distribution (Cui et al., 2023; Huang et al., 2023; Su et al., 2025) of toxic *Amanita*, there is a critical demand for rapid detection. In addition to the sensitivity, specificity, and speed of detection, other requirements must be satisfied; in a clinical context, the exact or most likely causative *Amanita* species is usually identified from a panel of suspected candidates showing similar symptoms with little retention of distinguishable morphology, such as color or shape. Moreover, strong compatibility with parallel detection and low dependence on instrumentation should also be taken into consideration. Based on these requirements, we conducted a proof-of-concept study to establish a multiplex detection system for toxic *Amanita* based on isothermal RPA and CRISPR/Cas12b technology.

Following the strategy validated in our earlier study (Li et al., 2025), candidate primer pairs and sgRNAs were designed for a panel of toxic *Amanita* species for isothermal RPA and CRISPR/Cas12b detection. The optimal combination of primer pair and sgRNA was then selected for each species based on the resulting fluorescence signal intensity. The target mushroom species could be specifically distinguished from non-target ones with the optimal assays. Simultaneously, the detection capacity was demonstrated in the range of 100 ng to 10 pg of genome DNA per reaction. Lastly, parallel detection was established using LFSs and a microfluidic disc chip, whose detection performance was validated with various DNA samples, including a mixed sample.

In this study, 10 pg of genome DNA per reaction was the lowest input sample dose tested. In preparation for this dosage, 100 ng of genomic DNA can be extracted from a few milligrams of dried mushrooms (Wang et al., 2017; Stefanova et al., 2022), which is sufficient to support approximately 10,000 reactions at the current dosage of DNA. Meanwhile, the lowest amount of template DNA used in the RPA-based assays is reportedly 1 pg per reaction for the *Mucorales* and *Paracoccidioides* spp (Xu et al., 2023; Lorenzini Campos et al., 2026). Thus, even when the biomass of toxic mushroom in a real sample is low, it is feasible to obtain DNA containing a sufficient quantity of mushroom-derived material to initiate the assays.

To achieve intuitive visualization of the results, LFSs were employed in conjunction with the RPA and CRISPR assays as inexpensive, easily fabricated, and widely used tools. A similar strategy has been employed for simple, rapid, and sensitive detection of *Mycoplasma hominis* (Chen et al., 2022), *Staphylococcus aureus* (Li Y. et al., 2022), duck adenovirus 3 (Liang et al., 2025), and *Trichomonas vaginalis* (Li S. et al., 2022). A threshold of 0.3 copies/μL of DNA was reached in the detection of duck adenovirus 3 (Liang et al., 2025). This suggests the potential for further development of the current assays to support the diagnosis of mushroom poisoning in the future.

To achieve a higher level of integration, a prototype microfluidic disc chip was developed with the established assays, which could be used to detect targets in a mixed sample clearly. Encouragingly, a similar strategy has been employed in the high-throughput diagnosis of various pathogens, such as *Vibrio parahaemolyticus* (Fang et al., 2026). In future developments, further integration can be achieved alongside the emergence of new assays for additional species, which would enable parallel detection of greater complexity.

However, given the proof-of-concept nature of this work, several limitations should be clarified and addressed in future studies.

First, only genomic DNA samples purified from authenticated mushroom specimens were used for validation in this work; no clinically relevant materials, such as gastric contents, vomit, or food matrices, were employed. Therefore, essential evaluations need to be performed to establish the actual performances of the assays with such complex biological matrices before field or clinical deployment.

Second, the specificity panel considered in this study comprised only seven toxic *Amanita* species; no edible mushrooms or morphologically similar non-toxic species were included. For future cross-reactivity tests, more species must be adopted to assess the true specificity in a real-world setting accurately, including edible *Amanita*, non-*Amanita*, and toxic mushroom species with or without relevance to the ones tested herein.

Third, the analytical detection limit of 10 pg per reaction was established from representative fluorescence curves. Additional independent replicates are still required at this and lower input amounts so that formal statistical characterizations, including confidence intervals, coefficient of variation, and inter-assay reproducibility, can be performed. In addition, receiver operating characteristic analysis should be conducted to evaluate the diagnostic accuracy before clinical use.

Fourth, the current microfluidic disc incorporated only four selected assays as a preliminary prototype to demonstrate proof-of-concept multiplex performance within the physical constraints of the disc, which limit the number, shapes, and volumes of the amplification chambers. This is a scalable design and does not represent a ceiling on the capacity of the system. Nevertheless, more extensive testing is necessary to demonstrate the performance of the microfluidic disc in combination with RPA-CRISPR assays for DNA mixtures of diverse ratios, species, and sources in a systematic manner.

Finally, it is also important to assess the stability of the lyophilized assays under different storage conditions, including temperature, humidity, and duration, so that their robustness can be established under field-relevant conditions.

## Data Availability

The original contributions presented in the study are included in the article/supplementary material, and further inquiries can be directed to the corresponding author.
